# The gap-free genome and multi-omics analysis of *Citrus reticulata* ‘Chachi’ reveal the dynamics of fruit flavonoid biosynthesis

**DOI:** 10.1093/hr/uhae177

**Published:** 2024-06-27

**Authors:** Congyi Zhu, Congjun You, Pingzhi Wu, Yongjing Huang, Ruimin Zhang, Zhengyan Fan, Chao Yu, Jinli Gong, Xiaoli Hu, Jiwu Zeng, Xuepeng Sun

**Affiliations:** Institute of Fruit Tree Research, Guangdong Academy of Agricultural Sciences, Key Laboratory of South Subtropical Fruit Biology and Genetic Resource Utilization (MOA), Guangdong Province Key Laboratory of Tropical and Subtropical Fruit Tree Research, Guangzhou 510640, China; Collaborative Innovation Center for Efficient and Green Production of Agriculture in Mountainous Areas of Zhejiang Province, College of Horticulture Science, Zhejiang A&F University, Hangzhou 311300, Zhejiang, China; Key Laboratory of Quality and Safety Control for Subtropical Fruit and Vegetable, Ministry of Agriculture and Rural Affairs, Zhejiang A&F University, Hangzhou 311300, Zhejiang, China; Institute of Fruit Tree Research, Guangdong Academy of Agricultural Sciences, Key Laboratory of South Subtropical Fruit Biology and Genetic Resource Utilization (MOA), Guangdong Province Key Laboratory of Tropical and Subtropical Fruit Tree Research, Guangzhou 510640, China; Institute of Fruit Tree Research, Guangdong Academy of Agricultural Sciences, Key Laboratory of South Subtropical Fruit Biology and Genetic Resource Utilization (MOA), Guangdong Province Key Laboratory of Tropical and Subtropical Fruit Tree Research, Guangzhou 510640, China; Institute of Fruit Tree Research, Guangdong Academy of Agricultural Sciences, Key Laboratory of South Subtropical Fruit Biology and Genetic Resource Utilization (MOA), Guangdong Province Key Laboratory of Tropical and Subtropical Fruit Tree Research, Guangzhou 510640, China; Institute of Fruit Tree Research, Guangdong Academy of Agricultural Sciences, Key Laboratory of South Subtropical Fruit Biology and Genetic Resource Utilization (MOA), Guangdong Province Key Laboratory of Tropical and Subtropical Fruit Tree Research, Guangzhou 510640, China; Collaborative Innovation Center for Efficient and Green Production of Agriculture in Mountainous Areas of Zhejiang Province, College of Horticulture Science, Zhejiang A&F University, Hangzhou 311300, Zhejiang, China; Key Laboratory of Quality and Safety Control for Subtropical Fruit and Vegetable, Ministry of Agriculture and Rural Affairs, Zhejiang A&F University, Hangzhou 311300, Zhejiang, China; Collaborative Innovation Center for Efficient and Green Production of Agriculture in Mountainous Areas of Zhejiang Province, College of Horticulture Science, Zhejiang A&F University, Hangzhou 311300, Zhejiang, China; Key Laboratory of Quality and Safety Control for Subtropical Fruit and Vegetable, Ministry of Agriculture and Rural Affairs, Zhejiang A&F University, Hangzhou 311300, Zhejiang, China; Collaborative Innovation Center for Efficient and Green Production of Agriculture in Mountainous Areas of Zhejiang Province, College of Horticulture Science, Zhejiang A&F University, Hangzhou 311300, Zhejiang, China; Key Laboratory of Quality and Safety Control for Subtropical Fruit and Vegetable, Ministry of Agriculture and Rural Affairs, Zhejiang A&F University, Hangzhou 311300, Zhejiang, China; Institute of Fruit Tree Research, Guangdong Academy of Agricultural Sciences, Key Laboratory of South Subtropical Fruit Biology and Genetic Resource Utilization (MOA), Guangdong Province Key Laboratory of Tropical and Subtropical Fruit Tree Research, Guangzhou 510640, China; Collaborative Innovation Center for Efficient and Green Production of Agriculture in Mountainous Areas of Zhejiang Province, College of Horticulture Science, Zhejiang A&F University, Hangzhou 311300, Zhejiang, China; Key Laboratory of Quality and Safety Control for Subtropical Fruit and Vegetable, Ministry of Agriculture and Rural Affairs, Zhejiang A&F University, Hangzhou 311300, Zhejiang, China

## Abstract

*Citrus reticulata* ‘Chachi’ (CRC) has long been recognized for its nutritional benefits, health-promoting properties, and pharmacological potential. Despite its importance, the bioactive components of CRC and their biosynthetic pathways have remained largely unexplored. In this study, we introduce a gap-free genome assembly for CRC, which has a size of 312.97 Mb and a contig N50 size of 32.18 Mb. We identified key structural genes, transcription factors, and metabolites crucial to flavonoid biosynthesis through genomic, transcriptomic, and metabolomic analyses. Our analyses reveal that 409 flavonoid metabolites, accounting for 83.30% of the total identified, are highly concentrated in the early stage of fruit development. This concentration decreases as the fruit develops, with a notable decline in compounds such as hesperetin, naringin, and most polymethoxyflavones observed in later fruit development stages. Additionally, we have examined the expression of 21 structural genes within the flavonoid biosynthetic pathway, and found a significant reduction in the expression levels of key genes including *4CL*, *CHS*, *CHI*, *FLS*, *F3H*, and *4′OMT* during fruit development, aligning with the trend of flavonoid metabolite accumulation. In conclusion, this study offers deep insights into the genomic evolution, biosynthesis processes, and the nutritional and medicinal properties of CRC, which lay a solid foundation for further gene function studies and germplasm improvement in citrus.

## Introduction

Citrus, cultivated primarily in tropical and subtropical regions, is among the most significant fruit crops, renowned for its delightful taste. While the peel of most citrus varieties is commonly discarded as inedible byproduct, the dried pericarp of *Citrus reticulata* ‘Chachi’ (CRC), notably sourced from Xinhui County in the Guangdong province of China, is widely used in cuisine and traditional Chinese medicine due to its unique flavor and health-promoting effects [1]. The medicinal history of CRC dates back over 2000 years to the Han Dynasty, documented in the ancient Chinese materia medica, ‘Shennong’s Classic of Materia Medica’ (also known as ‘Shen Nong Ben Cao Jing’) [2]. Beyond its historical roots, CRC continues to play a significant role in modern clinical practice, addressing conditions such as anepithymia, indigestion, abdominal fullness, distention, vomiting, and cough with expectoration of phlegm [3]. The most recent 2020 edition of the Chinese Pharmacopoeia lists CRC in 176 different prescriptions, constituting over 10% of Chinese patent medicines. Contemporary pharmaceutical investigations underscore the diverse pharmacological effects of CRC extract, including impacts on the gastrointestinal and respiratory systems, as well as exhibiting antioxidant, anti-inflammatory, and anticancer activities [4, 5].

Flavonoids and volatile oils constitute the principle bioactive constituents within CRC. The intricate repertoire of chemical compounds in CRC peel encompasses over 200 flavonoid metabolites, including flavonoid glycosides and polymethoxyflavones (PMFs) [6, 7]. Hesperidin, nobiletin, and tangeretin, which are recognized as index compounds within CRC, have been listed in the Chinese Pharmacopoeia. The composition and distribution of flavonoids exhibit notable variability, both temporally and spatially. For instance, studies have shown that flavonoid *O*-glycosides and *C*-glycosides, exemplified by naringenin, naringin, and prunin, reach peak concentrations at early stages of fruit development and diminish with fruit ripening [7, 8]. Conversely, certain PMFs, including tangeretin and nobiletin, exhibit higher concentrations in the immediate proximity to maturation, and mature samples display elevated levels of hesperetin and apigenin [9]. An association study between flavonoid content and antioxidant efficacy corroborates the premise that CRC harvested at different times is endowed with disparate therapeutic potentialities, which highlights the necessity to understand the biosynthesis and accumulation of bioactive components during fruit development of CRC.

Genes associated with flavonoid biosynthesis have continuously been identified, including transcription factors (TFs) that function as either positive or negative regulators, such as MBW complex components [10], AaYABBY5 [11], and MYBs [12]. For *Citrus grandis*, a comprehensive analyses of fruit transcriptome and metabolome data suggest the involvement of CgCHS, CgCHI, Cg4CL2, and Cg4CL3 in naringenin biosynthesis. Additionally, Cg1, 2RhaT and CgFNS are found to participate in biosynthesis of naringin and rhoifolin, respectively [13]. In sour orange (*Citrus aurantium*), CHS, CHI, CYP75B1, CYP81Q32, and nine other components exhibit a high correlation with flavonoid accumulation during fruit development [14]. Moreover, several uridine diphosphate (UDP)-sugar dependent glycosyltransferase (UGT) genes catalyzing flavonoid glycosylation have been reported in different citrus species, which include *Cm1,2RhaT*, *Cs1,6RhaT*, and *Crc1,6RhaT *[15–17]. Although recent studies have shown that MYB, C2C2-Dof, and Alfin-like TFs are likely involved in regulation of flavonoid biosynthesis in CRC [18], a comprehensive view of flavonoid composition and its regulation during fruit development is still lacking.

Since the genome sequencing of the first *Citrus* plant was completed in 2013 [19], high-quality genome assemblies have been achieved for ~20 cultivated and wild *Citrus* species [20]. Notably, a previous study produced a draft genome of the Mangshan wild mandarin (*C. reticulata*) from the Nanling Mountains of South China. This wild mandarin represents a primitive form of *C. reticulata*, which has been domesticated independently with substantial introgression from pummelos [21]. In this study, we present a high-quality gap-free genome assembly of a cultivated line of *C. reticulata* ‘Chachi’, namely Dazhongyoushen, which is widely cultivated in Guangdong province of China. This genome assembly, together with the transcriptomic and metabolomic profiles from four major stages of fruit development, provides new insights into flavonoid biosynthesis and nutritional properties of CRC fruit. The dataset provided in this study will also facilitate gene function analysis as well as germplasm improvement in future breeding programs.

## Results

### Genome assembly and annotation of CRC

**Figure 1:**
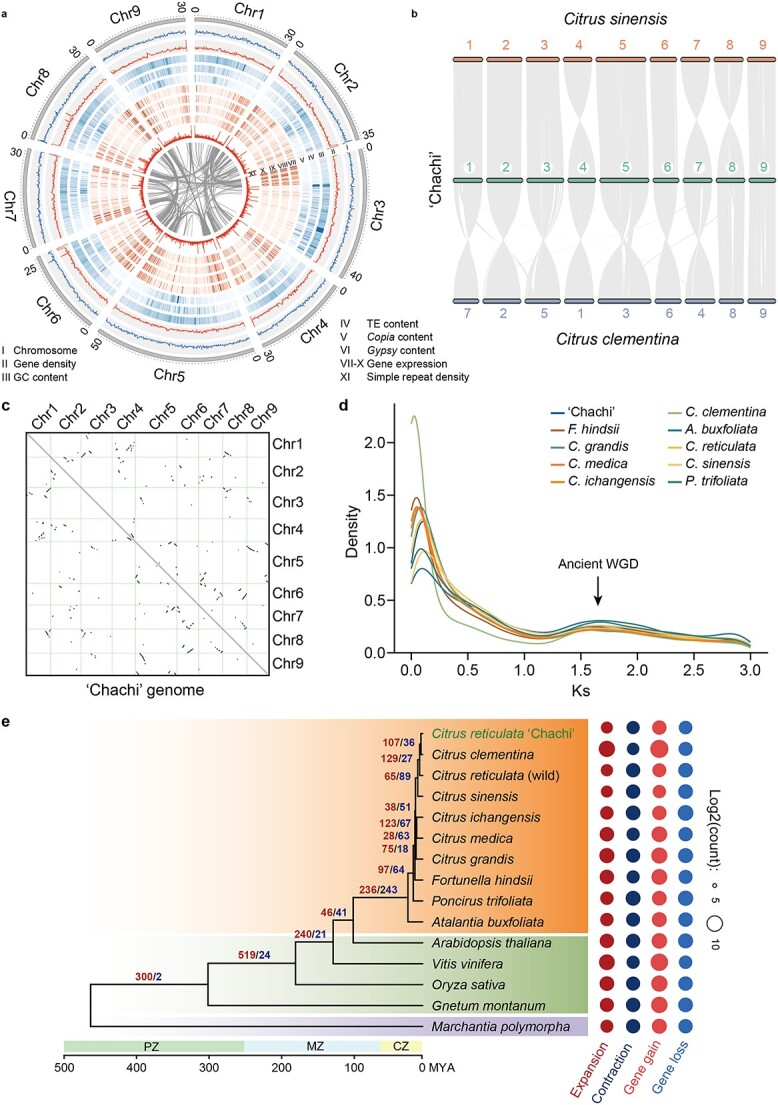
Comparative genomics of CRC and closely related species. **a** Genomic features of CRC. Each feature is summarized based on a 100-kb window. Tracks VII–X represent gene expression of fruit at SIM, IM, NM, and M stages, respectively. **b** Genome collinearity analysis of CRC, *Citrus sinensis*, and *Citrus clementina*. **c** Syntenic analysis within the genome of CRC. **d** Synonymous substitution rate (*K*_s_) of paralogs in CRC and other species. The peak indicates that an ancient whole-genome duplication (WGD) occurred in the common ancestor of *Citrus* species. **e** Gene family expansion (red) and contraction (blue) during the evolution of representative land plant species, including CRC. PZ, MZ, and CZ indicate the Paleozoic, Mesozoic, and Cenozoic eras, respectively. The orange background indicates *Citrus*-related species, while green indicates other seed plants, and purple indicates bryophytes. Numbers on branches are the sizes of expanded (blue) and contracted (red) gene families at each node at *P* < 0.05.

A total of 23.1 Gb nanopore long reads (N50 >40 kb) and 21.1 Gb nanopore ultralong reads (N50 >100 kb) were generated for the CRC genome (Supplementary Data Table S1). We compared the metrics of assemblies generated from each dataset, as well as a combination of both long and ultralong reads and found that the assembly derived from long reads alone showed overall higher contiguity than the others. This long read assembly was further polished by 29.4 Gb MGI short reads, followed by the removal of haplotigs. The final assembly consisted of 16 contigs, with a total size of 307.7 Mb and an N50 size of 30.2 Mb. To further improve the genome, we employed the ultralong read assembly from the same pipeline to scaffold the assembly, which yielded nine supercontigs, corresponding to the nine chromosomes of the genome. Consequently, the gap-free genome assembly reached a size of 312 974 977 bp and an N50 size of 32 187 089 bp, which closely approximates the estimated genome size of 315.6 Mb as determined by the *K*-mer analysis (Fig. 1a; Supplementary Data Fig. S1). This assembly exhibited significantly higher contiguity compared with the genome of the wild *C. reticulata*, which had a contig N50 size of 24 761 bp [21]. A genome-wide search for centromeric satellite repeats and telomeric repeats (CCCTAAA/TTTAGGG) uncovered the positions of centromeres on nine chromosomes, as well as telomeres at both ends of chromosomes 2, 5, and 6, and at one end of chromosomes 1, 4, 7, and 9 (Supplementary Data Fig. S2).

BUSCO [22] evolution showed that 98.4% of the broadly conserved genes were captured by the CRC assembly. The LTR assembly index (LAI) for this genome was 24.95, exceeding the benchmark proposed for a gold-standard reference genome [23]. Collinearity analysis indicated a high degree of synteny among the genomes of CRC, *Citrus sinensis* and *Citrus clementina* (Fig. 1b). In addition, the Merqury consensus quality value [24] for the assembly was 33, and the RNA read mapping rates were 95.33%. These metrics together indicated the high quality of the CRC genome assembly.

### Whole-genome duplication and phylogenetic evolution

To unravel the evolutionary history of the CRC, we analyzed the synonymous substitution rates (*K*_s_) among paralogs within this genome and compared them with those in other previously studied citrus species. We detected a clear peak of *K*s values between 1 and 2, with a summit around 1.6 for all citrus genomes, suggesting that an ancient whole-genome duplication took place in the common ancestor of citrus species (Fig. 1c and d). Phylogenetic analysis involving CRC and an additional 14 plant species based on 411 single-copy orthogroups positioned CRC in close relation to *C. clementina*, both of which belong to modern mandarins (Fig. 1e). Gene family evolution analysis revealed that 132 gene families (comprising 1157 genes) have significantly expanded (*P* < 0.05) in the CRC genome compared with its last common ancestor with *C. clementina*, including those encoding UDP-glycosyltransferase, shikimate *O*-hydroxycinnamoyltransferase, and phenylalanine ammonia-lyase, which are critical components of flavonoid biosynthesis (Supplementary Data Table S2).

### Dynamics of flavonoid metabolism during fruit development of CRC

**Figure 2:**
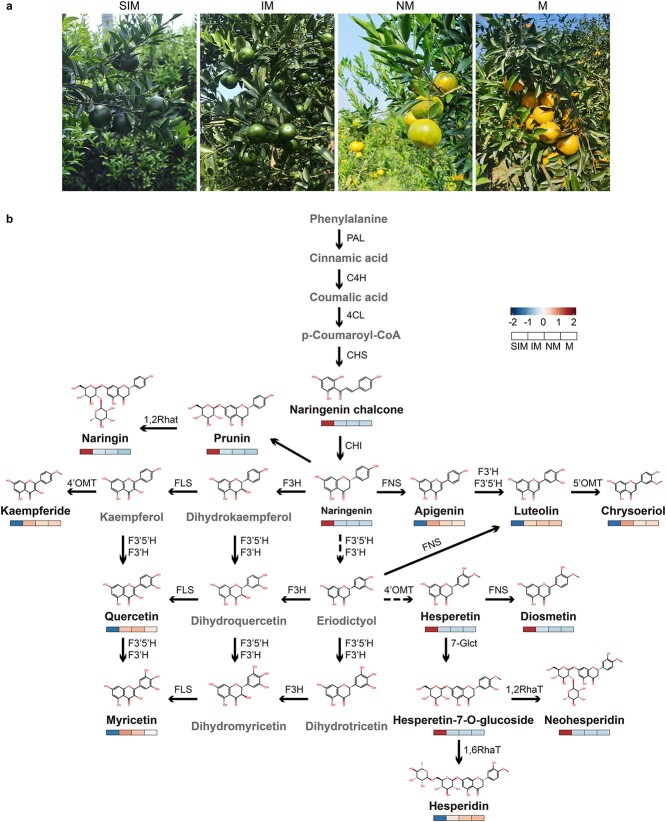
Metabolic profiles of CRC fruits at four representative developmental stages. **a** Fruit of CRC at small immature stage (SIM), immature stage (IM), near mature stage (NM), and mature stage (M). **b** Flavonoid biosynthetic pathways and the concentration of the metabolites during fruit development. The heat map shows the normalized average concentration of each metabolite in the fruit at different developmental stages. Metabolites undetected or absent in CRC fruit are indicated with gray names.

Flavonoids constitute the primary active components in CRC peels. To study when and how these metabolites were synthesized and accumulated, we sampled the fruit peels at four distinct developmental stages, including the small immature stage (SIM), immature stage (IM), near-mature stage (NM), and mature stage (M) (Fig. 2a). A comprehensive analysis using UPLC-MS-based untargeted metabolomics identified 491 flavonoid metabolites in the peel of CRC fruit at four stages, among which flavones were the most diverse, comprising 243 compounds (49.49%), followed by flavonols with 115 compounds (23.42%) (Supplementary Data Table S3; Supplementary Data Fig. S3). The metabolic profiles of flavonoids were highly reproducible between biological replicates but were remarkably dynamic among fruits at different stages (Supplementary Data Fig. S4a). In general, the majority of flavonoid metabolites (83.3%; *n* = 409) exhibited high concentrations in the early developmental stages of fruit and declined during the course of fruit maturation (Supplementary Data Table S3; Supplementary Data Fig. S4b). These included key metabolites of the flavonoid biosynthesis pathway, such as chalcone and naringenin, both of which showed a high concentration in small immature fruit but were undetectable in the subsequent three stages, as well as four flavanones (i.e. prunin, naringin, hesperetin, and hesperetin-7-*O*-glucoside) that were progressively reduced by 65.49–98.96% during fruit development (Fig. 2b; Supplementary Data Table S3). In contrast, the terminal products of the pathway, including three flavones (apigenin, luteolin, and chrysoeriol) and three flavonols (quercetin, myricetin, and kaempferide), showed the opposite trend and accumulated in mature fruit. Moreover, the concentration of hesperidin, a primary bioactive compound in CRC fruit, was increased by 1.73-, 1.96-, and 2.05-fold in IM, NM, and M fruits, respectively, compared with that in the SIM fruit (Fig. 2b).

In addition, we identified 76 PMFs in the peel of CRC fruit, including 14 dimethoxyflavones, 20 trimethoxyflavones, 17 tetramethoxyflavones, 16 pentamethoxyflavones, 8 hexamethoxyflavones, and 1 heptamethoxyflavone. Among these, 45 (59.21%) PMF compounds were present in SIM fruit but were almost undetectable in the subsequent developmental stages (Supplementary Data Fig. S5). Likewise, sinensetin was most abundant in SIM fruit, followed by a gradual decrease during fruit maturation, reaching a minimum at the mature stage with a 76.97% reduction. The contents of nobiletin, tangeretin, and eight other PMF compounds also declined markedly with fruit development, while being almost stable in NM and M fruit (Supplementary Data Table S3). Conversely, retusin was the most abundant PMF compound in the IM, NM, and M fruit, but barely detectable in the SIM fruit (Supplementary Data Table S3; Supplementary Data Fig. S5).

Most flavonoids can be glycosylated. Based on the detected glycosylation patterns in our data, flavonoids can be categorized into several groups according to their modification groups. *O*-Glycosylation commonly occurred at position 3 or 7, while *C*-glycosylation was observed at either position 6 or 8. There were also di-glycosylated flavonoids (Supplementary Data Table S3). Among the naringenin derivatives ever reported, five were detected. Narirutin (naringenin-7-*O*-rutinoside) and naringin (naringenin-7-*O*-neohesperidoside) showed significantly higher levels compared with the other three glycosylated naringenin compounds. Hesperetin primarily occurred as hesperetin-7-*O*-glucoside, followed by hesperidin (hesperetin-7-*O*-rutinoside) and neohesperidin (hesperetin-7-*O*-neohesperidoside). The major kaempferol glycoside was 3-*O*-glycosylation. Among the kaempferol derivatives, kaempferol-3-*O*-neohesperidoside exhibited the highest abundance, constituting 19.04–25.83% of the total amount of kaempferol derivatives at different fruit development stages. This was followed by kaempferol-3-*O*-glucorhamnoside and kaempferol-3-*O*-glucoside-7-*O*-rhamnoside. Quercetin derivatives underwent *O*-glycosylation at positions 3 and 7. The glycosylated derivatives of quercetin showed the highest content in SIM fruit and decreased as the fruit developed. The total content of quercetin 3-*O*-glycosides was 1.39–1.79 times higher than that of quercetin 7-*O*-glycosides in fruit at four stages. In addition to *O*-glycosylation at position 7, three flavones (apigenin, luteolin, and chrysoeriol) underwent *C*-glycosylation at position 6 or 8, as well as di-*C*-glycosylation. The peel of CRC fruit contained higher levels of isovitexin (apigenin-6-*C*-glucoside), isoorientin (luteolin-6-*C*-glucoside), orientin (luteolin-8-C-glucoside), and scoparin (chrysoeriol-8-*C*-glucoside) at the SIM stage. However, these compounds were present in trace amounts during the other three fruit developmental stages. Notably, the contents of chrysoeriol-6,8-di-*C*-glucoside in IM, SM, and M fruit were 36.39-, 30.30-, and 32.64-fold higher, respectively, than that in SIM fruit (Supplementary Data Table S3).

### Transcriptional regulation of flavonoid biosynthesis

**Figure 3:**
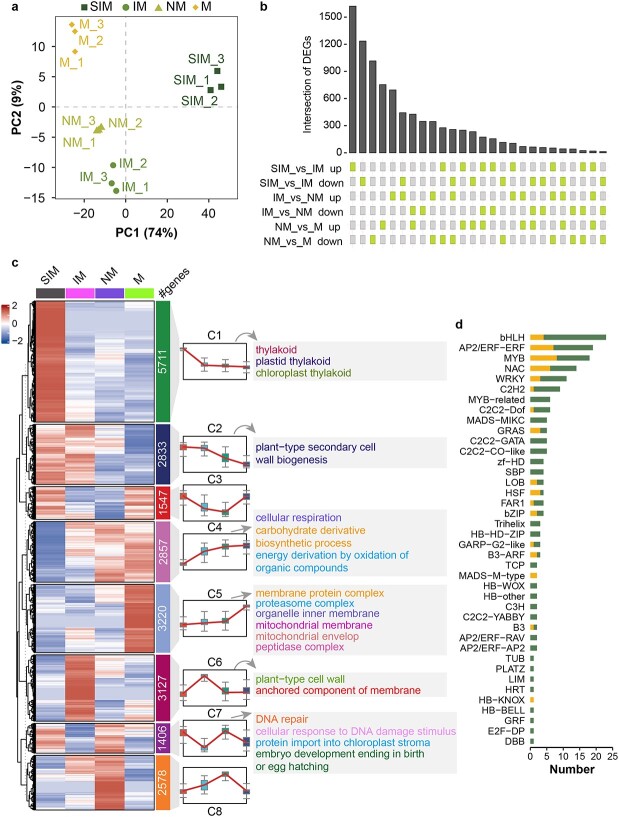
Transcriptional dynamics of CRC fruit at four stages. **a** Principal component analysis of fruit samples based on gene expression data. **b** Upset diagram showing the intersection of DEGs in different contexts of comparison. **c***K*-means clustering analysis of genes based on their expression data. **d** Classification and the of numbers of differentially expressed transcription factors in the comparison between SIM and M.

We performed transcriptome sequencing using the same samples as the metabolomics analysis. PCA analysis separated the samples in a manner similar to the pattern observed in metabolomics, suggesting that transcriptional regulation dominated metabolic flux in CRC fruit (Fig. 3a; Supplementary Data Fig. S4a). We identified 1621 genes upregulated and 1235 genes downregulated in the SIM versus IM comparison. The number of differentially expressed genes (DEGs) decreased in subsequent stages, with 754 upregulated and 1016 downregulated genes in the NM versus M comparison, and 659 upregulated and 348 downregulated genes in the IM versus NM comparison (Fig. 3b). *K*-Means clustering of samples based on gene expression revealed eight significant gene groups (Fig. 3c), among which the largest group (C1) was enriched with genes associated with chloroplast functions. Genes within this group were expressed at higher levels in SIM fruit than in other fruits. Conversely, groups showing gene expression positively correlated with fruit development (C4 and C5) were predominantly associated with mitochondrial functions (Fig. 3c). These data were consistent with the fact that fruit ripening was accompanied by the breakdown of chloroplasts and an increase in respiration rate. Furthermore, 189 (8.31%) of DEGs were identified as TFs (Fig. 3d), and most of them were downregulated (*n* = 141).

We identified 21 genes in the CRC genome that potentially participate in flavonoid biosynthesis, based on homology searches with genes from other species (Supplementary Data Fig. S6a). The biosynthesis begins with the phenylpropanoid pathway, which involves three major enzymes: phenylalanine ammonia lyase (PAL), cinnamic acid 4-hydroxylase (C4H), and 4-coumarate:CoA ligase (4CL). Subsequently, *p*-coumaroyl-CoA is converted to multiple intermediates and end-products through a series of enzyme activities, including chalcone synthase (CHS), chalcone isomerase (CHI), flavone synthase (FNS), flavanone 3-hydroxylase (F3H), flavonoid 3′-monooxygenase (F3′H), flavonoid-3′,5′-hydroxylase (F3′5′H), flavonol synthase (FLS), 4′-*O*-methyltransferase (4′OMT), and 5′-*O*-methyltransferase (5′OMT). Evolutionary analysis indicated that most of these 21 genes were under purifying selection (Supplementary Data Table S4), while expression analysis revealed distinct expression patterns for these genes during fruit development (Supplementary Data Fig. S6a). Four putative PAL genes were detected in all four stages of fruit development. Among these four genes, the *CP7g002665* gene was highly expressed in SIM, IM, and M stages and, interestingly, it was significantly lower by 40.53% in the NM stage compared with its highest expression level at the M stage. The expression trend of the *CP6g002191* gene was similar to that of the *CP7g002665* gene. The expression of *CP8g002821* was significantly lower than the other three putative PAL genes. For the two putative C4H genes, the expression of the *CP1g000830* gene increased with fruit development, reaching its highest expression at the M stage. CHS genes, encoding an important rate-limiting enzyme in flavonoid biosynthesis, converted *p*-coumaroyl-CoA to naringenin chalcone. Two CHS genes (*CP2g002894* and *CP1g000339*), identified in the CRC genome, exhibited distinct expression patterns. The highest expression of *CP2g002894* was observed at the SIM stage, and it decreased by 71, 66, and 80% at the IM stage, NM stage, and M stage, respectively. However, *CP1g000339* showed a rising–declining trend during fruit development, with the lowest expression level at the SIM stage and the highest at the IM stage. Additionally, the expression levels of *FLS1*, *F3H*, and *7-Glct* genes were similar to that of *CP2g002894*. 4′OMT is a critical methyltransferase that catalyzes the transfer of a methyl group to eriodictyol at the 4′ position of the B ring, converting it to hesperetin. The expression level of the gene encoding 4′OMT (*CP9g001415*) decreased gradually during fruit development, with the highest expression at the SIM stage. On the other hand, the expression levels of *F3′5′H* and *F3′H* genes showed an increasing trend during fruit development, with the lowest expression at the SIM stage. Overall, our analysis revealed distinctive expression patterns of key enzymes in the flavonoid biosynthetic pathway during fruit development of CRC, suggesting their potential roles in regulating flavonoid accumulation.

Moreover, we conducted a correlation analysis using a dataset comprising 21 genes and 18 metabolites across the four stages of fruit development (Supplementary Data Fig. S6b). Within the phenylpropanoid pathway, it was observed that four PAL genes lacked a consistent and obvious correlation with the majority of flavonoids. However, two 4CL genes, *CP5g001726* and *CP4g000284*, demonstrated a positive correlation with several flavonoids, including naringenin chalcone, naringenin, hesperetin, diosmin, and neohesperidin. Interestingly, these genes were negatively correlated with other flavonoids, such as hesperidin, kaempferide, quercetin, myricetin, and chrysoeriol. Naringenin chalcone, the product catalyzed by the CHS enzyme, was positively correlated with *CP2g002894* (*r* = 0.96) and negatively with *CP1g000339* (*r* = −0.85). The gene *CP7g002886*, which encodes a CHI enzyme, showed a strong positive correlation with both its substrate (naringenin chalcone) and product (naringenin), with correlations exceeding 0.90. Moreover, a significant positive correlation (*r* = 0.92) was found between the expression of 4′OMT and its product, hesperetin.

### Identification of gene modules related to flavonoid biosynthesis

**Figure 4:**
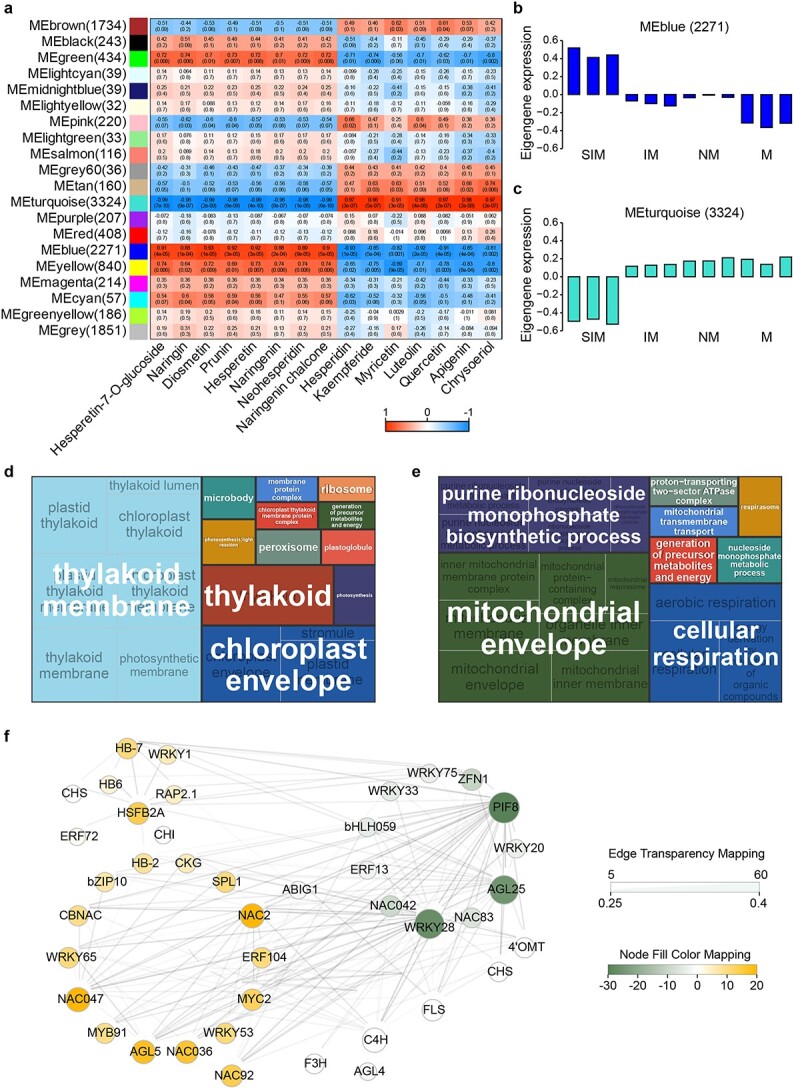
WGCNA analysis of CRC transcriptomes. **a** Relationship between gene co-expression modules and contents of selected metabolites. Numbers in each cell represent a correlation coefficient and a *P* value (within parentheses). **b**, **c** Expression pattern of the blue and turquoise modules. The *x*-axis shows replicated transcriptome samples from four developmental stages; the *y*-axis indicates the eigengene value. Gene number of each module is shown on the top. **d**, **e** Enriched GO terms of the blue (**d**) and turquoise (**e**) module genes. **f** Regulatory network of TFs and flavonoid biosynthetic genes. Dot sizes and colors represent the numbers of correlated genes.

To explore the gene regulatory network governing flavonoid synthesis in the peel of CRC fruit, we utilized a weighted gene co-expression network analysis (WGCNA) with transcriptome and metabolome data. This yielded 20 distinct co-expression modules (Fig. 4a), each assigned a unique color and correlated with flavonoid content. Notably, two module eigengenes (MEs) were found to significantly correlate (*P* < 0.05) with 15 flavonoids. The turquoise module, encompassing 3324 genes, exhibited strong positive correlations with hesperidin (*r* = 0.97), kaempferide (*r* = 0.96), luteolin (*r* = 0.98), quercetin (*r* = 0.97), apigenin (*r* = 0.98), and chrysoeriol (*r* = 0.97). Conversely, significant negative correlations were observed with hesperetin-7-*O*-glucoside (*r* = −0.99), genistein (*r* = −0.97), naringin (*r* = −0.96), and others. The blue module, containing 2271 genes, showed an opposite pattern of correlation to the turquoise module, with the highest positive correlations with flavonoids such as hesperetin-7-*O*-glucoside and genistein, and negative correlations with hesperidin, kaempferide, and others. Structural genes of the flavonoid biosynthetic pathway, including *CHS*, *FLS*, and *4′OMT*, were highly expressed in the blue module during the SIM stage, aligning with flavonoid content variations (Fig. 4b). GO enrichment analysis linked the blue module to photosynthesis and plant development processes (Fig. 4d), while the turquoise module was associated with cellular respiration and energy generation (Fig. 4c and e).

TFs were integral to the network, with 399 TFs distributed across the flavonoid co-expression modules, predominantly from gene families like NAC, WRKY, bHLH, bZIP, and others, with nearly equal distribution between the turquoise and blue modules (Fig. 4f). Key TFs in the blue module were identified as positively related to several flavonoids, with PIF8 and AGL25 showing extensive connectivity. In the turquoise module, TFs such as AGL5 and NAC2 exhibited strong positive correlations with specific flavonoids, while 14 TFs from various families were identified as negatively associated with flavonoid biosynthesis. Structural genes involved in flavonoid biosynthesis, like *C4H*, *CHS*, *CHI*, *FLS*, and *4′OMT*, showed co-expression with multiple TFs, highlighting a complex network of interactions facilitating flavonoid biosynthesis. This comprehensive analysis underscores the intricate regulatory mechanisms influencing flavonoid synthesis in CRC, critical for fruit development and quality.

### Characterization of UDP-glycosyltransferase in CRC

**Figure 5:**
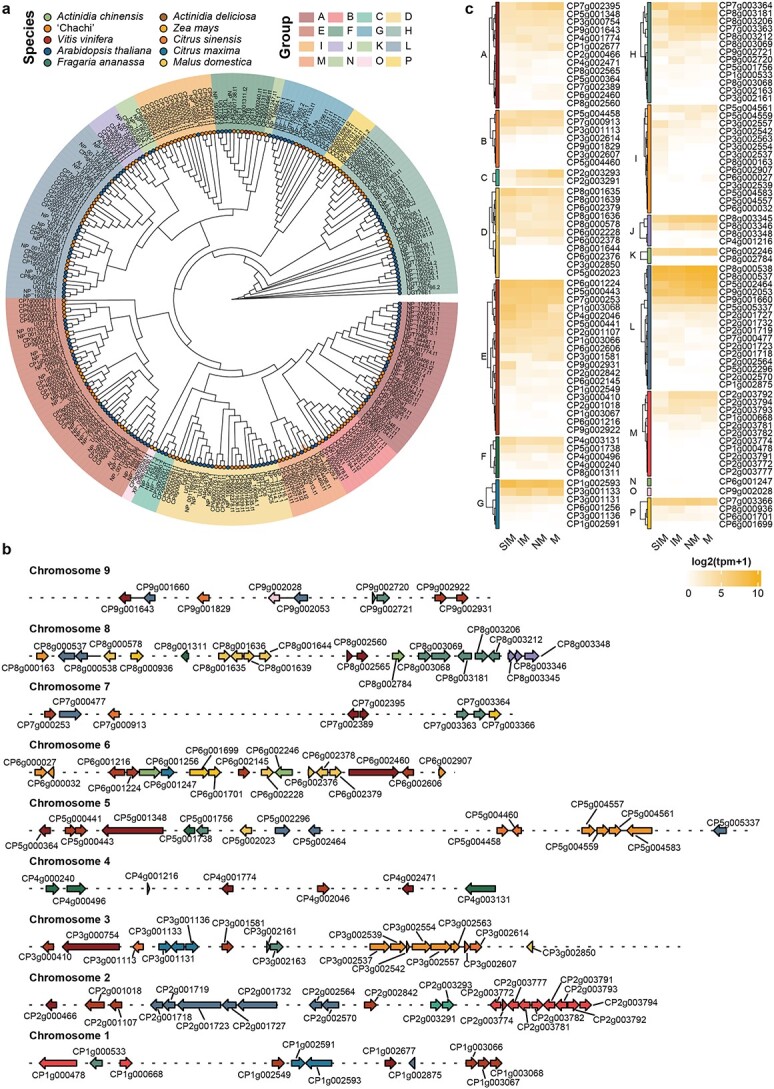
Analysis of UGT gene families in the CRC genome. **a** Maximum likelihood phylogeny of UGT genes in CRC and other selected plant species. **b** Chromosomal location of UGT genes in the CRC genome. **c** Expression profile of UGT genes in CRC fruit.

Flavonoids in plants are often glycosylated. We identified 129 UGTs in the CRC genome, containing a conserved plant secondary product glycosyltransferase (PSPG) domain at the C-terminus of the protein (Fig. 5a). The number of UGT genes in CRC was significantly higher than that in model plants such as *Arabidopsis thaliana* (*n* = 107), but lower than in apple (*n* = 241) and grape (*n* = 181). The UGT genes were unevenly distributed across the genome, with chromosomes 2, 3, and 8 harboring nearly half of the UGT genes (Fig. 5b). Intron mapping of the UGT genes revealed that 55 UGTs (43%) lacked introns, 48 UGTs (38%) contained one intron, and 11 UGTs (9%) contained two introns, while the remaining 14 UGTs contained more than two introns. To evaluate the evolutionary relationship of the UGT gene family, a phylogenetic tree was constructed based on *A. thaliana* UGTs and previously reported UGT genes in other species. The UGTs in CRC were divided into 16 groups, named Group A to Group P following the common naming rules of UGT genes. In CRC, Group E had the highest number of UGTs (*n* = 19), followed by Groups L, I, A, and H, each containing more than 12 members.

Gene expression of UGTs in the peel of CRC at the four stages was analyzed. Out of the total UGTs, 82 (63.5%) genes were expressed at one or more stages of fruit development (TPM [transcripts per million] >1) (Fig. 5c). Among these, more than 34 UGTs showed the highest transcript levels in SIM fruit, with 10, 6, and 5 UGT genes belonging to Groups E, D, and I, respectively.

## Discussion

This study reports a gap-free genome assembly for the ‘Chachi’ variety of *C. reticulata* (CRC), featuring a contig N50 size of 32 187 089 bp and highlighting its genetic complexity and evolutionary significance. CRC, recognized for its dual role as a food and medicinal plant, contains a plethora of biologically active substances. Prior research has largely focused on a limited range of compounds within CRC. Advanced techniques, such as HPLC fingerprinting, rapid-resolution LC-ESIMS and reverse-phase HPLC, have been used to identify and quantify characteristic components, including various flavonoids, in CRC and related species [25–27]. Despite these advancements, traditional methods often provide limited insights into a small number of bioactive compounds of CRC.

Metabolomics has emerged as a pivotal technology for identifying and quantifying various metabolites in medicinal herbs. Leveraging a state-of-the-art, widely targeted metabolomics approach, this study employed UPLC-ESI-MS/MS in conjunction with multiple reaction monitoring (MRM) scan mode for an expansive analysis of the metabolic profiles of CRC fruit peels. This comprehensive approach facilitated the identification of numerous metabolites, including various flavonoids, offering a more extensive investigation of CRC metabolites than previously reported. Our analysis revealed distinct accumulation patterns of specific metabolites across four developmental stages of the fruit. Notably, naringenin chalcone and several flavanones exhibited the highest concentrations during the early, SIM stage, with a progressive decrease as the fruit developed. This pattern suggests the SIM stage as critical for flavonoid biosynthesis, with subsequent fruit growth and ripening leading to a reduction in flavonoid content due to dilution, cell division, and increased catabolism over synthesis. Contrastingly, hesperidin, a bioactive compound extensively studied for its health benefits, showed a pattern of increasing concentration reaching its peak at the M stage. Similarly, the content trends of three flavonoids (apigenin, luteolin, and chrysoeriol) and four flavonols (quercetin, myricetin, kaempferide, and dihydrokaempferide) mirrored that of hesperidin. PMFs, unique to citrus, including tangeretin, nobiletin, and sinensetin, exhibited a gradual decrease from the SIM stage to fruit ripening. Retusin remained consistent in later developmental stages but was absent at the early stage. These findings align with previous research indicating rapid changes in flavonoid content during CRC fruit ripening. It should be noted that although the metabolic changes between SIM and IM are significant, our sampling may have missed the critical transition point in metabolite content after the SIM stage. Therefore, more frequent sampling is required in the future to uncover a clearer trend of metabolic changes in CRC fruit during development.

The diverse structures of flavonoids correlate with their varied pharmacological properties. For instance, hesperidin is recognized for its neuroprotective and antidiabetic effects, apigenin for its anti-adipogenesis activity, quercetin for its anti-inflammatory, immunomodulatory, and anti-oxidative properties, and myricetin for its broad spectrum of health benefits, including immunomodulatory, analgesic, antimicrobial, anti-oxidant, antidiabetic, anticancer, and antihypertensive effects. PMFs like nobiletin and tangeretin, which decrease through the stages of CRC fruit development, have shown potential in anti-oxidative, anticardiovascular disease, and anticancer therapies. Moreover, specific PMFs present at the SIM stage demonstrated significant anti-inflammatory and anti-obesity activities. This study underscores the importance of harvesting CRC fruit at various stages to maximize the medicinal benefits of its metabolites, providing a scientific foundation for their informed and rational use.

The biosynthetic pathway of flavonoids has been well documented, with the identification of key structural genes encoding major enzymes across various plant species. This study integrates genomic, transcriptomic, and metabonomic analyses to shed light on the biosynthesis of flavonoids in CRC, and identifies several critical genes. Notably, the genes encoding CHS, CHI, F3H, and FLS displayed the highest expression levels at the SIM stage, with a subsequent decline in expression. CHS, the initial enzyme in flavonoid biosynthesis, directs the phenylpropanoid pathway towards flavonoid production. Two CHS genes were identified in CRC fruit, exhibiting distinct expression patterns; one maintained low expression throughout fruit development, whereas the other was significantly upregulated at the SIM stage, suggesting a predominant role in flavonoid biosynthesis. In citrus, the biosynthesis of flavor-determining non-bitter flavanone-7-*O*-rutinosides and bitter flavanone-7-*O*-neohesperidosides is catalyzed by 1,6-rhamnosytransferase (1,6RhaT) and 1,2-rhamnosytransferase (1,2RhaT), respectively. Our findings reveal a homolog to the 1,6RhaT gene in CRC, with its highest expression at the SIM stage, aligning with previous studies on its expression in other citrus fruits. The integrative analysis of metabolome and transcriptome provided interesting insights into the flavonoid biosynthetic pathway in CRC. In the upstream portion, the changes in gene expression and metabolite content were consistent. For example, the expression of the *CHS* and *CHI* genes positively correlated with their products, naringenin chalcone and naringenin, respectively. However, discrepancies between gene expression and metabolite levels were primarily observed in the downstream pathway. A notable example was the *FLS* gene and its direct product, quercetin. Despite the highest expression level of the *FLS* gene occurring at the SIM stage – 3.60-, 2.66-, and 4.61-fold greater than the other three fruit development stages – the quercetin content at the SIM stage was significantly lower than at the other stages. Conversely, the content of major quercetin glycosides (e.g. quercetin-3-*O*-robinobioside, quercetin-3-*O*-glucoside, and quercetin-7-*O*-rutinoside) at the SIM stage was ~10-fold higher than at the other stages. Thus, when considering the derivatives of flavonoids, such as glycosylates, the changes in synthase gene expression and metabolite production in flavonoid biosynthesis were largely consistent. Moreover, regulation of flavonoid biosynthesis involves the interplay of MYB and bHLH TFs, which were differentially expressed during fruit development. WGCNA analysis identified specific modules associated with hesperidin accumulation, indicating the regulatory roles of these TFs in flavonoid biosynthesis.

While previous research has illuminated the roles of numerous TFs in flavonoid biosynthesis, the intricacies of regulatory mechanisms within this pathway are still not fully comprehended in CRC. The WRKY TFs, one of the most expansive TF families, play critical roles in various plant processes, including growth, development, metabolism, responses to environmental stresses, and senescence. Studies in apples have shown that overexpressing MdWRKY11 and MdWRKY75 leads to increased accumulation of flavonoids and anthocyanins. Specifically, MdWRKY11 upregulates genes such as F3H, FLS, DFR, ANS, and UFGT, contributing to flavonoid production, while MdWRKY75 interacts with the promoters of MYB TFs, which are known not only for regulating anthocyanin synthesis but also for controlling anthocyanin transport. In our investigation, the bHLH, AP2/ERF-ERF, and MYB TF families were identified as the most prominently differentially expressed TFs across all DEGs when comparing the SIM stage versus the M stage. Subsequent analysis through WGCNA helped establish a gene-to-metabolite correlation network, pinpointing WRKY28 and PIF8 as significantly associated with several key structural genes in flavonoid biosynthesis, including *C4H*, *CHS*, *FLS*, and *FSH*. The temporal expression patterns of these two newly identified TFs suggest their potential involvement in regulating flavonoid biosynthesis in CRC, marking a step forward in unraveling the complex regulatory network governing this critical metabolic pathway.

## Materials and methods

### Sample collection

Leaf samples of CRC were obtained from the citrus germplasm resource nursery in Guangzhou city, Guangdong province, located at a longitude of 113.3712° and a latitude of 23.1504°. Fruit samples of CRC were collected from Sanjiang town in the Xinhui district of Jiangmen city, with coordinates of longitude 113.1061° and latitude 22.4679°. Fruits were harvested from three distinct trees in late July (SIM stage), early November (IM stage), late November (NM stage), and mid-December (M stage) of year 2020. Each sampling contained at least six fruits, and all samples were immediately frozen in liquid nitrogen and stored at −80°C until usage.

### Genome sequencing, assembly, and functional annotation

The genome was sequenced using the Oxford Nanopore long-read sequencing platform. NextDenovo software [28] (v2.5.2) was employed with default parameters to assemble the long reads. Subsequently, the assembly underwent polishing with NextPolish [29] (v1.4.1), utilizing both nanopore long reads and MGI short reads as inputs. The polished assembly was processed with purge_dups [30] to remove haplotigs and then scaffolded with a second assembly derived from ultralong reads. Transposable elements were identified using the EDTA package [31]. Gene prediction was performed with BRAKER2 [32]. The coverage of gene space was assessed using BUSCO [22]. Functional annotation of predicted genes was performed by comparing with the KEGG, TrEMBL, and GenBank databases under the E-value threshold of 1E−5. GO annotation was carried out by InterProScan [33]. The TFs were identified by iTAK [34] with default parameters. Telomere and centromere analyses were performed using the quarTeT pipeline [35].

### Phylogeny and whole-genome duplication analysis

For phylogenetic analysis, protein sequences were clustered into orthogroups using OrthoFinder [36] with default parameters. MAFFT [37] was employed to align protein sequences, and trimAl [38] was used to trim the alignments. The resulting aligned sequences were concatenated, and a maximum likelihood phylogeny among species was constructed using IQ-TREE2 [39] with 1000 bootstraps. The divergence times were estimated using the MCMCtree module of the PAML package [40], and fossil ages used for tree calibration were obtained from a previous publication [41]. Gene family expansion and contraction were analyzed using CAFE5 [42] with a *P*-value threshold of 0.05. For whole-genome duplication analysis, an all-against-all BLASTp search was conducted using Diamond [43] with default settings. These alignments were then utilized to identify syntenic gene pairs in collinear blocks with JCVI (https://github.com/tanghaibao/jcvi). The Yn00 module of the PAML package was utilized to calculate synonymous substitutions per synonymous site (*K*_s_).

### Identification of flavonoid biosynthetic genes

Protein sequences of CRC were used to identify candidate genes encoding key enzymes involved in the flavonoid biosynthetic pathway under the cutoff of E-value ≤1e−5 and identity ≥40%. Query sequences for the BLAST search comprised all known CHS and CHI genes in *C. sinensis*, as well as those encoding potential flavonol synthase, flavanone 3-hydroxylase, flavonoid 3′-hydroxylase, *O*-methyltransferase, and glycosyltransferase, from diverse sources such as *Glycine max* [44], *Citrus unshiu* Marc [45], *Oryza sativa* [46], *Citrus grandis* [47], and *Cajanus cajan* [48].

### Quantification of flavonoids

Samples were prepared and extracted for metabolomic analysis following an established protocol [49]. A composite sample, made up of aliquots from each fruit, was used to evaluate the stability and repeatability of the untargeted metabolomics method. The extracted samples were analyzed using a UPLC-ESI-MS/MS system comprising a Shim-pack UFLC Shimadzu CBM30A system for UPLC and an Applied Biosystems 6500 QTRAP for MS. The UPLC conditions included using an Agilent SB-C18 column (1.8 μm, 2.1 mm × 100 mm), an injection volume of 4 μl, a column temperature of 40°C, a flow rate of 0.35 ml/min, and a solvent system of acetonitrile and water. The gradient program was set to start at 95:5 v/v at 0 min, shifting to 5:95 v/v at 9.0 min, maintained at 5:95 v/v through 10.0 min, and returning to 95:5 v/v at 11.1 min, holding until 14.0 min. The UHPLC effluent was then directly introduced into an ESI-triple QTRAP-MS system. Optimized ESI settings included a turbo spray ion source at a temperature of 550°C, ion spray voltages of 5.5/−4.5 kV, and gas pressures of 50 psi for gas I, 60 psi for gas II, and 25 psi for the curtain gas. QQQ scans were performed in multiple reaction monitoring (MRM) mode, with each scan optimized for de-clustering potential (DP) and collision energy (CE).

For flavonoid quantification, all MS data were utilized to annotate metabolites based on the in-house database and widely recognized public metabolite databases such as MassBank, HMDB, ChemBank, PubChem, and METLIN. Chromatographic peak areas of corresponding metabolites across different samples were corrected and integrated for quantitative analysis using MultiQuant software. Statistical analyses, including PCA, correlation coefficient, and hierarchical clustering, were conducted using R software. The ropls module in R was employed for partial least squares-discriminant analysis (PLS-DA). Significant differences in metabolite levels between groups were identified based on a variable importance in projection (VIP) of at least 1, an absolute log_2_ fold change (FC) of ≥1, and a *P*-value of <0.05.

### Transcriptome sequencing and analysis

Total RNA was extracted from samples using TRIzol reagent, adhering to the manufacturer’s instructions. RNA-Seq library construction and sequencing were entrusted to a specialized transcriptome sequencing company (Majorbio, Shanghai, China). Subsequently, 12 libraries were sequenced (NovaSeq 6000, Illumina) under paired-end sequencing mode (2 × 150 bp). The raw reads were cleaned and then aligned to the CRC genome using HISAT2 software [50]. Transcript expression levels were quantified using RSEM software [51], measured as transcripts per million (TPM). DEG analysis between two samples was conducted using DESeq2 [52], with expression differences defined as |log_2_FC| > 1 and adjusted *P* value ≤0.05. *K*-means clustering analysis was carried out using the clusterGVis module in the R package.

### Phylogenetic analysis of UGT family

UGTs were predicted using hmmsearch against the PF00201 domain with default parameters. Subsequently, these predicted genes were verified in the SMART database to confirm the completeness of the conserved 44-amino-acid PSPG box. Protein sequences of UGTs were aligned using MAFFT software and then trimmed with trimAl. The maximum likelihood phylogeny was constructed by IQ-TREE2 with 1000 bootstrap replicates.

### Gene network analysis

The WGCNA R package was used for network analysis. A dataset comprising 16 932 genes, each with >15 counts in 75% of the samples, was employed for the co-expression network analysis. The settings for the analysis included a soft threshold power of 16, a minimum module size of 30, and a cutHeight of 0.25. Consequently, 11 844 genes were categorized into 15 tissue-specific modules, while 5088 genes were identified as outliers and grouped into the gray module. Eigengene values for each module were computed and tested for associations with metabolic data. GO enrichment analysis for the module genes was visually represented using TreeMap and REVIGO [53]. Each rectangle in the visualization represented a single cluster representative; these were combined into superclusters of loosely related terms, differentiated by color. The size of each rectangle was proportional to the significance of the *P*-value. The resulting co-expression network was visualized using Cytoscape [54].

## Supplementary Material

Web_Material_uhae177

## Data Availability

The genome assembly and raw reads from genome and transcriptome sequencing have been submitted to the China National GeneBank DataBase (CNGBdb) under accession number CNP0005504. The genome assembly and its annotation are also accessible on Figshare: https://figshare.com/articles/dataset/Genome_and_annotation_of_i_Citrus_reticulata_i_Chachi_/25470037.

## References

[1] Zheng GD , Hu PJ, Chao YX. et al. Nobiletin induces growth inhibition and apoptosis in human nasopharyngeal carcinoma C666-1 cells through regulating PARP-2/SIRT1/AMPK signaling pathway. *Food Sci Nutr*. 2019;7:1104–12 30918653 10.1002/fsn3.953 PMC6418462

[2] Liang PL , Chen XL, Gong MJ. et al. Guang Chen Pi (the pericarp of *Citrus reticulata* Blanco’s cultivars ‘Chachi’) inhibits macrophage-derived foam cell formation. *J Ethnopharmacol*. 2022;293:115328 35489660 10.1016/j.jep.2022.115328

[3] Yu X , Sun S, Guo Y. et al. Citri Reticulatae Pericarpium (Chenpi): botany, ethnopharmacology, phytochemistry, and pharmacology of a frequently used traditional Chinese medicine. *J Ethnopharmacol*. 2018;220:265–82 29628291 10.1016/j.jep.2018.03.031

[4] Zheng YY , Zeng X, Peng W. et al. Characterisation and classification of Citri Reticulatae Pericarpium varieties based on UHPLC-Q-TOF-MS/MS combined with multivariate statistical analyses. *Phytochem Anal*. 2019;30:278–91 30588683 10.1002/pca.2812

[5] Zheng Y , Zeng X, Chen P. et al. Integrating pharmacology and gut microbiota analysis to explore the mechanism of Citri Reticulatae Pericarpium against reserpine-induced spleen deficiency in rats. *Front Pharmacol*. 2020;11:586350 33192528 10.3389/fphar.2020.586350 PMC7606944

[6] Zhang N , Wu W, Huang Y. et al. Citrus flavone tangeretin inhibits CRPC cell proliferation by regulating Cx26, AKT, and AR signaling. *Evid Based Complement Alternat Med*. 2022;2022:1–15 10.1155/2022/6422500 PMC8803427 35111229

[7] Liang S , Zhang J, Liu Y. et al. Study on flavonoids and bioactivity features of pericarp of *Citrus reticulata* “Chachi” at different harvest periods. *Plan Theory*. 2022;11:3390 10.3390/plants11233390 PMC9737822 36501428

[8] Saini RK , Ranjit A, Sharma K. et al. Bioactive compounds of citrus fruits: a review of composition and health benefits of carotenoids, flavonoids, limonoids, and terpenes. *Antioxidants*. 2022;11:239 35204122 10.3390/antiox11020239 PMC8868476

[9] Peng Z , Zhang H, Li W. et al. Comparative profiling and natural variation of polymethoxylated flavones in various citrus germplasms. *Food Chem*. 2021;354:129499 33752115 10.1016/j.foodchem.2021.129499

[10] Xu P , Wu L, Cao M. et al. Identification of MBW complex components implicated in the biosynthesis of flavonoids in woodland strawberry. *Front*. *Plant Sci*. 2021;12:774943 10.3389/fpls.2021.774943 PMC8606683 34819941

[11] Kayani SI , Shen Q, Rahman SU. et al. Transcriptional regulation of flavonoid biosynthesis in *Artemisia annua* by AaYABBY5. *Hortic Res*. 2021;8:257 34848710 10.1038/s41438-021-00693-x PMC8632904

[12] Liu GX , Jiang Y. et al. UV-B promotes flavonoid biosynthesis in *Ginkgo biloba* by inducing the GbHY5-GbMYB1-GbFLS module. *Hortic Res*. 2023;10:uhad118 37547729 10.1093/hr/uhad118 PMC10402656

[13] Fan R , Zhu C, Qiu D. et al. Integrated transcriptomic and metabolomic analyses reveal key genes controlling flavonoid biosynthesis in *Citrus grandis* ‘Tomentosa’ fruits. *Plant Physiol Biochem*. 2023;196:210–21 36724705 10.1016/j.plaphy.2023.01.050

[14] Chen J , Shi Y, Zhong Y. et al. Transcriptome analysis and HPLC profiling of flavonoid biosynthesis in *Citrus aurantium* L. during its key developmental stages. *Biology*. 2022;11:1078 36101454 10.3390/biology11071078 PMC9313048

[15] Frydman A , Weisshaus O, Bar-Peled M. et al. Citrus fruit bitter flavors: isolation and functional characterization of the gene Cm1,2RhaT encoding a 1,2 rhamnosyltransferase, a key enzyme in the biosynthesis of the bitter flavonoids of citrus. *Plant J*. 2004;40:88–100 15361143 10.1111/j.1365-313X.2004.02193.x

[16] Liu X , Lin C, Ma X. et al. Functional characterization of a flavonoid glycosyltransferase in sweet orange (*Citrus sinensis*). *Front*. *Plant Sci*. 2018;9:166 10.3389/fpls.2018.00166 PMC5818429 29497429

[17] Shang N , Tong P, Ye P. et al. Crc1,6RhaT is involved in the synthesis of hesperidin of the main bioactive substance in the *Citrus reticulata* ‘Chachi’ fruit. *Hortic Plant J*. 2023;

[18] Su J , Peng T, Bai M. et al. Transcriptome and metabolome analyses provide insights into the flavonoid accumulation in peels of *Citrus reticulata* ‘Chachi’. *Molecules*. 2022;27:6476 36235014 10.3390/molecules27196476 PMC9570620

[19] Xu Q , Chen LL, Ruan X. et al. The draft genome of sweet orange (*Citrus sinensis*). *Nat Genet*. 2013;45:59–66 23179022 10.1038/ng.2472

[20] Huang Y , He J, Xu Y. et al. Pangenome analysis provides insight into the evolution of the orange subfamily and a key gene for citric acid accumulation in citrus fruits. *Nat Genet*. 2023;55:1964–75 37783780 10.1038/s41588-023-01516-6

[21] Wang L , He F, Huang Y. et al. Genome of wild mandarin and domestication history of mandarin. *Mol Plant*. 2018;11:1024–37 29885473 10.1016/j.molp.2018.06.001

[22] Simão FA , Waterhouse RM, Ioannidis P. et al. BUSCO: assessing genome assembly and annotation completeness with single-copy orthologs. *Bioinformatics*. 2015;31:3210–2 26059717 10.1093/bioinformatics/btv351

[23] Ou S , Chen J, Jiang N. Assessing genome assembly quality using the LTR assembly index (LAI). *Nucleic Acids Res*. 2018;46:e126 30107434 10.1093/nar/gky730 PMC6265445

[24] Rhie A , Walenz BP, Koren S. et al. Merqury: reference-free quality, completeness, and phasing assessment for genome assemblies. *Genome Biol*. 2020;21:245 32928274 10.1186/s13059-020-02134-9 PMC7488777

[25] Yi LZ , Yuan DL, Liang YZ. et al. Fingerprinting alterations of secondary metabolites of tangerine peels during growth by HPLC-DAD and chemometric methods. *Anal Chim Acta*. 2009;649:43–51 19664461 10.1016/j.aca.2009.07.009

[26] Liu EH , Zhao P, Duan L. et al. Simultaneous determination of six bioactive flavonoids in Citri Reticulatae Pericarpium by rapid resolution liquid chromatography coupled with triple quadrupole electrospray tandem mass spectrometry. *Food Chem*. 2013;141:3977–83 23993574 10.1016/j.foodchem.2013.06.077

[27] Li T , Li X, Zhang M. et al. Development and validation of RP-HPLC method for the simultaneous quantification of seven flavonoids in Pericarpium Citri Reticulatae. *Food Anal Methods*. 2014;7:89–99

[28] Hu, Wang Z, Sun Z. et al. An efficient error correction and accurate assembly tool for noisy long reads. *bioRxiv*. 2023. 10.1186/s13059-024-03252-4 PMC11046930 38671502

[29] Hu J , Fan J, Sun Z. et al. NextPolish: a fast and efficient genome polishing tool for long-read assembly. *Bioinformatics*. 2020;36:2253–5 31778144 10.1093/bioinformatics/btz891

[30] Guan D , McCarthy SA, Wood J. et al. Identifying and removing haplotypic duplication in primary genome assemblies. *Bioinformatics*. 2020;36:2896–8 31971576 10.1093/bioinformatics/btaa025 PMC7203741

[31] Ou S , Su W, Liao Y. et al. Benchmarking transposable element annotation methods for creation of a streamlined, comprehensive pipeline. *Genome Biol*. 2019;20:1–18 31843001 10.1186/s13059-019-1905-y PMC6913007

[32] Brůna T , Hoff KJ, Lomsadze A. et al. BRAKER2: automatic eukaryotic genome annotation with GeneMark-EP+ and AUGUSTUS supported by a protein database. *NAR Genom Bioinform*. 2021;3:lqaa108 33575650 10.1093/nargab/lqaa108 PMC7787252

[33] Jones P , Binns D, Chang HY. et al. InterProScan 5: genome-scale protein function classification. *Bioinformatics*. 2014;30:1236–40 24451626 10.1093/bioinformatics/btu031 PMC3998142

[34] Zheng Y , Jiao C, Sun H. et al. iTAK: a program for genome-wide prediction and classification of plant transcription factors, transcriptional regulators, and protein kinases. *Mol Plant*. 2016;9:1667–70 27717919 10.1016/j.molp.2016.09.014

[35] Lin Y , Ye C, Li X. et al. quarTeT: a telomere-to-telomere toolkit for gap-free genome assembly and centromeric repeat identification. Hortic Res. 2023;10:uhad127 37560017 10.1093/hr/uhad127 PMC10407605

[36] Emms DM , Kelly S. OrthoFinder: phylogenetic orthology inference for comparative genomics. *Genome Biol*. 2019;20:1–14 31727128 10.1186/s13059-019-1832-y PMC6857279

[37] Katoh K , Misawa K, Kuma KI. et al. MAFFT: a novel method for rapid multiple sequence alignment based on fast Fourier transform. *Nucleic Acids Res*. 2002;30:3059–66 12136088 10.1093/nar/gkf436 PMC135756

[38] Capella-Gutiérrez S , Silla-Martínez JM, Gabaldón T. trimAl: a tool for automated alignment trimming in large-scale phylogenetic analyses. *Bioinformatics*. 2009;25:1972–3 19505945 10.1093/bioinformatics/btp348 PMC2712344

[39] Minh BQ , Schmidt HA, Chernomor O. et al. IQ-TREE 2: new models and efficient methods for phylogenetic inference in the genomic era. *Mol Biol Evol*. 2020;37:1530–4 32011700 10.1093/molbev/msaa015 PMC7182206

[40] Yang Z . PAML 4: phylogenetic analysis by maximum likelihood. *Mol Biol Evol*. 2007;24:1586–91 17483113 10.1093/molbev/msm088

[41] Jiao C , Sørensen I, Sun X. et al. The *Penium margaritaceum* genome: hallmarks of the origins of land plants. *Cell*. 2020;181:1097–1111.e12 32442406 10.1016/j.cell.2020.04.019

[42] Mendes FK , Vanderpool D, Fulton B. et al. CAFE 5 models variation in evolutionary rates among gene families. *Bioinformatics*. 2020;36:5516–8 10.1093/bioinformatics/btaa1022 33325502

[43] Buchfink B , Reuter K, Drost HG. Sensitive protein alignments at tree-of-life scale using DIAMOND. *Nat Methods*. 2021;18:366–8 33828273 10.1038/s41592-021-01101-x PMC8026399

[44] Noguchi A , Saito A, Homma Y. et al. A UDP-glucose:isoflavone 7-O-glucosyltransferase from the roots of soybean (*Glycine max*) seedlings: purification, gene cloning, phylogenetics, and an implication for an alternative strategy of enzyme catalysis. *J Biol Chem*. 2007;282:23581–90 17565994 10.1074/jbc.M702651200

[45] Moriguchi T , Kita M, Ogawa K. et al. Flavonol synthase gene expression during citrus fruit development. *Physiol Plant*. 2002;114:251–8 11903972 10.1034/j.1399-3054.2002.1140211.x

[46] Falcone Ferreyra ML , Emiliani J, Rodriguez EJ. et al. The identification of maize and *Arabidopsis* type I FLAVONE SYNTHASEs links flavones with hormones and biotic interactions. *Plant Physiol*. 2015;169:1090–107 26269546 10.1104/pp.15.00515 PMC4587447

[47] Chen J , Li G, Zhang H. et al. Primary bitter taste of citrus is linked to a functional allele of the 1,2-rhamnosyltransferase gene originating from *Citrus grandis*. *J Agric Food Chem*. 2021;69:9869–82 34410124 10.1021/acs.jafc.1c01211

[48] Du T , Fan Y, Cao H. et al. Transcriptome analysis revealed key genes involved in flavonoid metabolism in response to jasmonic acid in pigeon pea (*Cajanus cajan* (L.) Millsp.). *Plant Physiol Biochem*. 2021;168:410–22 34715566 10.1016/j.plaphy.2021.10.022

[49] Kumar D , Ladaniya MS, Gurjar M. et al. Quantification of flavonoids, phenols and antioxidant potential from dropped *Citrus reticulata* Blanco fruits influenced by drying techniques. *Molecules*. 2021;26:4159 34299432 10.3390/molecules26144159 PMC8306461

[50] Kim D , Paggi JM, Park C. et al. Graph-based genome alignment and genotyping with HISAT2 and HISAT-genotype. *Nat Biotechnol*. 2019;37:907–15 31375807 10.1038/s41587-019-0201-4 PMC7605509

[51] Li B , Dewey CN. RSEM: accurate transcript quantification from RNA-Seq data with or without a reference genome. *BMC Bioinformatics*. 2011;12:1–16 21816040 10.1186/1471-2105-12-323 PMC3163565

[52] Love M , Anders S, Huber W. Differential analysis of count data – the DESeq2 package. *Genome Biol*. 2014;15:10–1186

[53] Supek F , Bošnjak M, Škunca N. et al. REVIGO summarizes and visualizes long lists of gene ontology terms. *PLoS One*. 2011;6:e21800 21789182 10.1371/journal.pone.0021800 PMC3138752

[54] Shannon P , Markiel A, Ozier O. et al. Cytoscape: a software environment for integrated models of biomolecular interaction networks. *Genome Res*. 2003;13:2498–504 14597658 10.1101/gr.1239303 PMC403769

