# Supplementary material for: The gap-free genome and multi-omics analysis of Citrus reticulata ‘Chachi’ reveal the dynamics of fruit flavonoid biosynthesis
Source: Hortic Res. 2024 Jun 27;11(8):uhae177. doi: 10.1093/hr/uhae177 (PMC11301317; doi:10.1093/hr/uhae177)
Supplement: Web_Material_uhae177 [file web_material_uhae177.zip › Supplementary_Figure3.pdf]

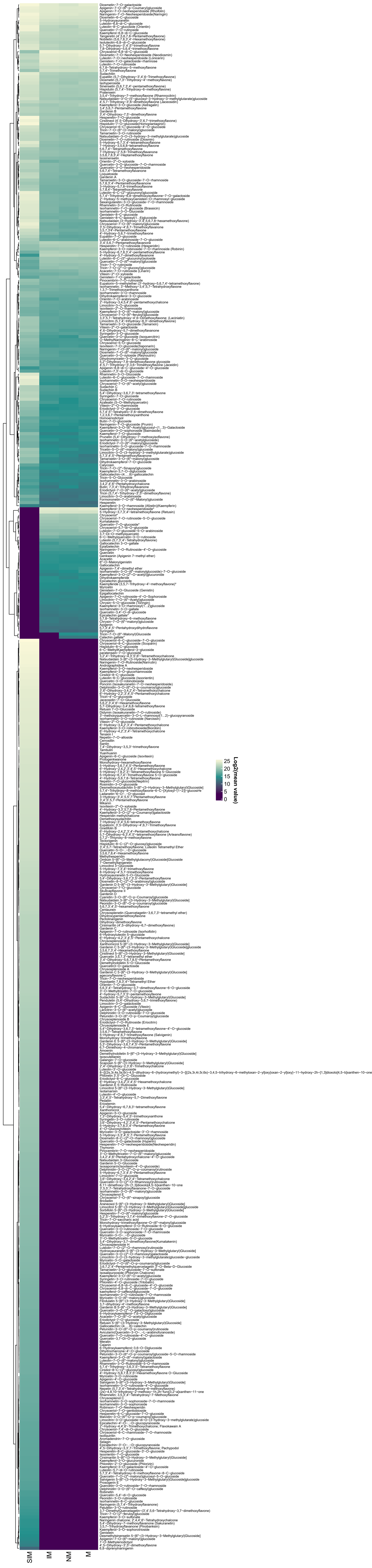
Log2(mean value)
25
20
15
10
5
0
SIM
IM
NM
M
