# Supplementary material for: The gap-free genome and multi-omics analysis of Citrus reticulata ‘Chachi’ reveal the dynamics of fruit flavonoid biosynthesis
Source: Hortic Res. 2024 Jun 27;11(8):uhae177. doi: 10.1093/hr/uhae177 (PMC11301317; doi:10.1093/hr/uhae177)
Supplement: Web_Material_uhae177 [file web_material_uhae177.zip › Supplementary_Figures_excludingS3.pdf]

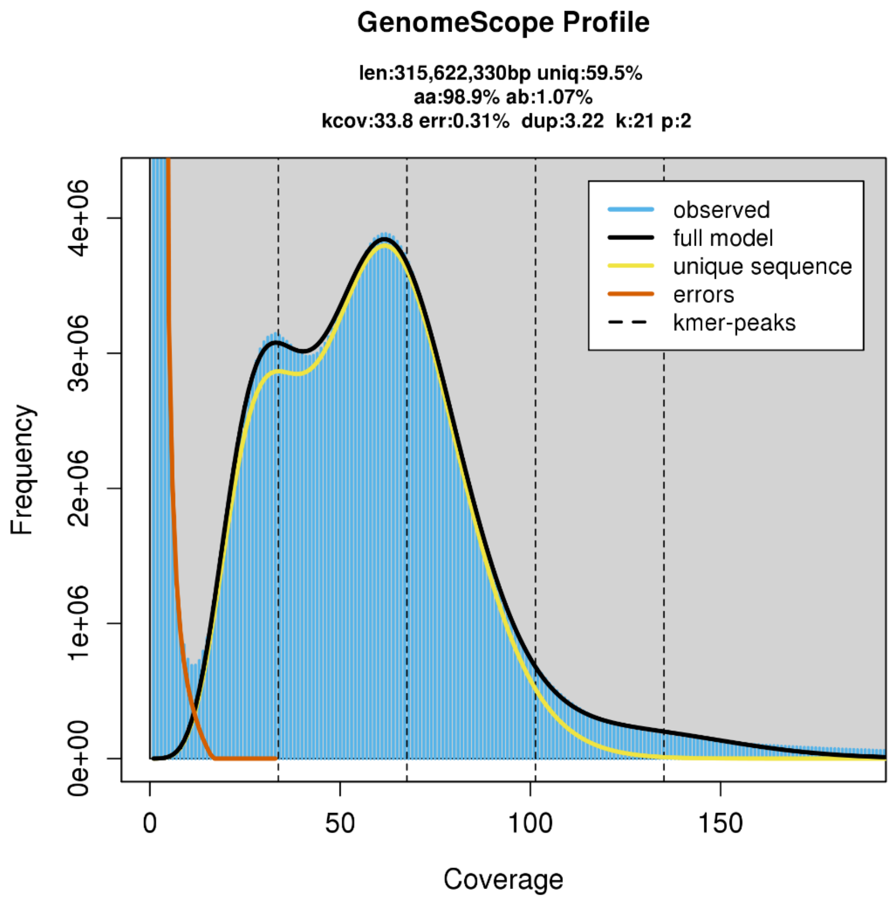

Supplementary Figure 1 The 21-mer spectrum of the short-read libraries.

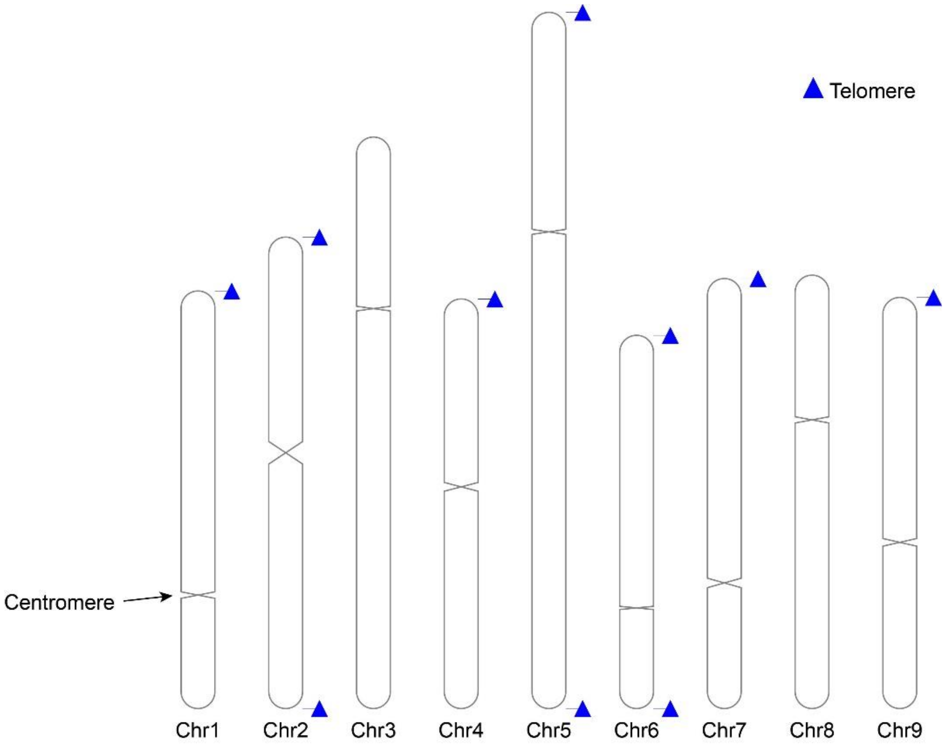

Supplementary Figure 2 Telomeres and centromeres identified in the CRC genome.

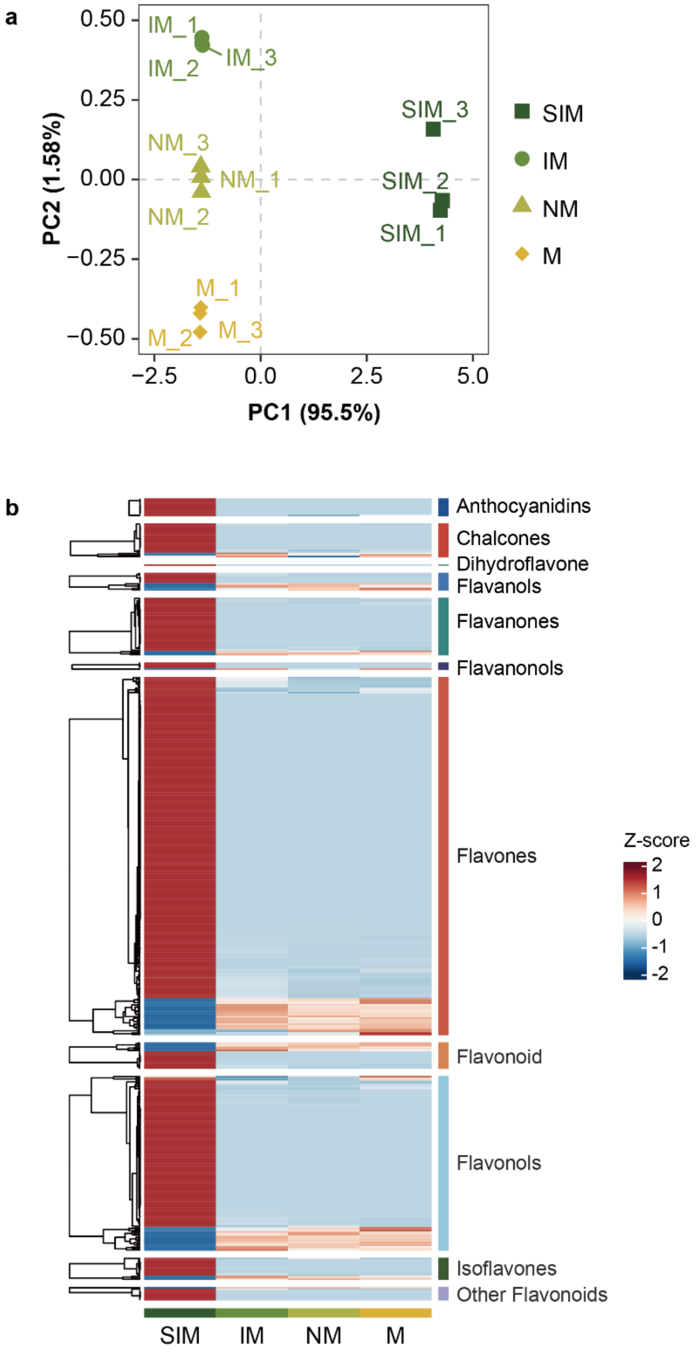

Supplementary Figure 4 Metabolic profiles of CRC fruit at four developmental stages. (**a**) PCA analysis of CRC fruit based on metabolic data. (**b**) The concentration of major flavonoids in CRC fruit during development.

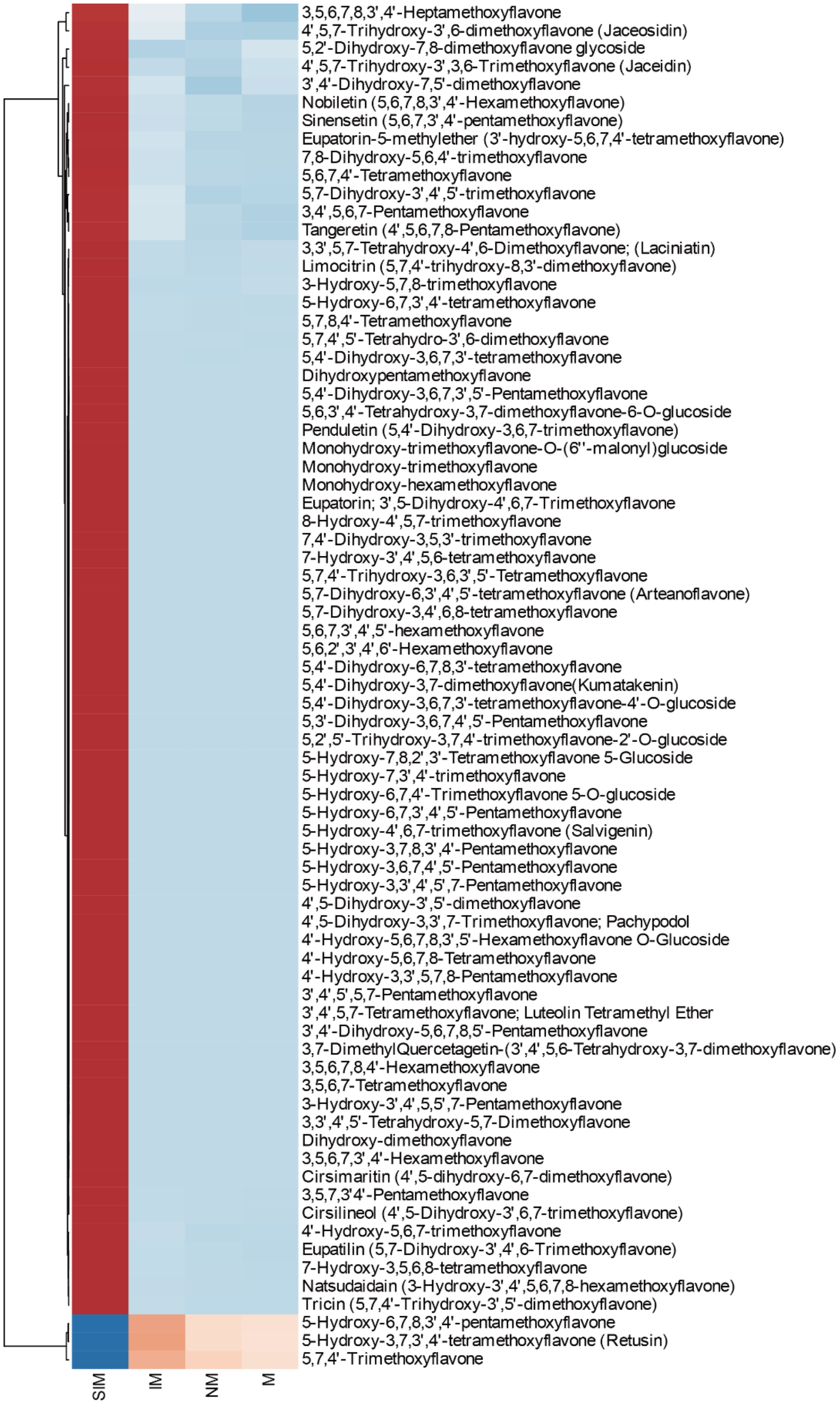

Supplementary Figure 5 The concentration of polymethoxyflavones in CRC fruit during development.

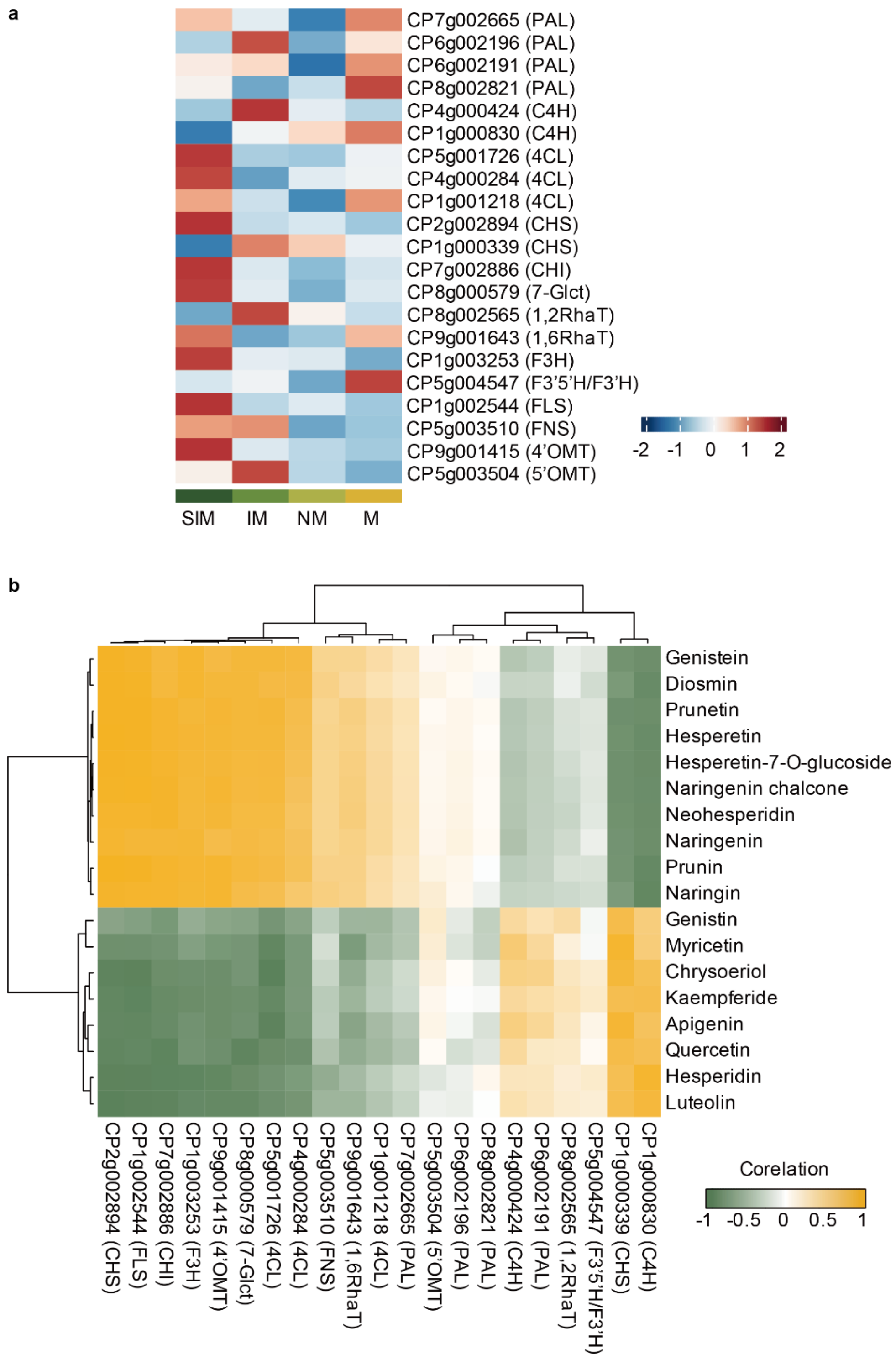

Supplementary Figure 6 Expression of genes involved in flavonoid biosynthesis. (**a**) Expression of flavonoid biosynthesis genes. (**b**) Correlation of gene expression and the concentration of metabolites.
